# 

**Published:** 1898-10

**Authors:** 


					﻿Southern Medical Record
A Monthly Journal of Medicine and Surgery.
Vol. XXVIII. ATLANTA, GA., OCTOBER, [1898.1 J }> R
BERNARD WOLFF, M. D., Editor.
Associate Editors :
A. W. GRIGGS, M. D.
j. McFadden gaston, Jr., m. d.
j. mcfadden gaston, m.d.
WILLIS r. WESTMORELAND, M. JD..
B. M. ZETTLER, Business Manager.
Entered at the Post-office at Atlanta, Ga., as Second-class Matter.
				

